# Identifying an Ectopic Parathyroid Adenoma Using 4DCT in a Pediatric Patient with Persistent Primary Hyperparathyroidism

**DOI:** 10.1155/2013/676039

**Published:** 2013-12-05

**Authors:** Vaninder K. Dhillon, Pisit Pitukcheewanont, Michael Yeh, Dennis Maceri

**Affiliations:** ^1^Department of Otolaryngology-Head and Neck Surgery, University of Southern California School of Medicine, 1200 North State Street GNH 4136, Los Angeles, CA 90033, USA; ^2^Department of Pediatric Endocrinology, Children's Hospital, Los Angeles, CA 90027, USA; ^3^Department of Endocrinology, University of California Los Angeles, Los Angeles, CA 90095, USA; ^4^Department of Pediatric Otolaryngology, Children's Hospital, Los Angeles, CA 90027, USA

## Abstract

We present a case of an ectopic mediastinal parathyroid adenoma detected with 4-dimensional computed tomography (4DCT) in a patient with persistent primary hyperparathyroidism and failed neck exploration. We discuss the utility of 4DCT in the localization of these lesions and offer an algorithm that implements the use of 4DCT early on when standard imaging techniques are nonlocalizing.

## 1. Introduction

Minimally invasive and directed extirpation of parathyroid lesions is the goal for patients with primary hyperparathyroidism (PHPT). Current guidelines for preoperative planning include imaging studies, such as a sestamibi scan, ultrasound, or MRI, to localize the adenoma and direct parathyroid surgery. These studies are not useful for patients who have ectopic glands, those located in the mediastinum, retroesophageal space, carotid sheath, thymus, or the thyroid. Roy et al. reported the incidence of occult parathyroid adenomas to be 22%; however, the range of ectopic adenomas has been shown to be from 6 to 16% [1]. For those patients who have failed neck exploration and have nonlocalizing studies, there is a need to accurately detect these lesions in order to decrease morbidity for these patients. 4D computed tomography (CT) has been shown to be useful in detecting these lesions, with a higher sensitivity and specificity than sestamibi scans, ultrasound, and MRI.

We present a case report of a pediatric patient with PHPT whose mediastinal parathyroid adenoma was detected on 4-dimensional computed tomography (4DCT) after failed neck explorations and negative ultrasound, sestamibi, and MR imaging. We review the role and technique of 4DCT and provide an algorithm by which to incorporate this new technology in a systematic fashion so that patients with persistent PHPT avoid increased morbidity with a second operation.

## 2. Case Presentation

A twelve-and-a-half-year-old boy was referred for primary hyperparathyroidism to our institution for second opinion for his hypercalcemia. His symptoms included fatigue and muscle pains, and routine laboratory values showed hypercalcemia by his pediatrician, so he was referred for an endocrinology evaluation. His only significant medical history was that he had sustained a right hand fracture the year before. MEN-1 gene testing was performed and was negative. Given his hypercalcemia, the assessment by one endocrinologist was parathyroid adenoma versus four-gland hyperplasia and the patient was referred to ENT for surgical evaluation.

One primary ENT surgeon assumed care of the patient upon referral. Imaging workup began with a thyroid ultrasound and a sestamibi, which did not demonstrate a parathyroid adenoma in the usual juxtathyroid locations. The standard of care for neck parathyroid adenomas is ultrasound and sestamibi of the neck alone in order to initially localize disease [1]. Given no localization, the patient underwent a four-gland exploration. Intraoperative findings showed two left sided normal parathyroids and a right inferior parathyroidectomy. Pathology of this lesion was consistent with parathyroid hyperplasia on permanent pathology. Postoperatively; however, he remained hypercalcemic.

The patient underwent a second neck exploration six months later after a repeat ultrasound showed a suspicious nodule in the right carotid sheath. At that surgery, venous intraoperative PTH sampling was performed which showed elevated levels in the left innominate vein and superior vena cava. Biochemical and radiologic evidence was conflicting. Even after this second exploration, postoperatively the patient continued to experience hypercalcemia and was maintained on Sensipar and 1,25-OH vitamin D. Despite medical therapy, his serum PTH and serum calcium levels remained elevated.

An MRI was performed which showed T2 hyperintensity left of the aortic arch, which led to the decision to obtain a 4DCT of the neck and mediastinum.

**Figure 1:**
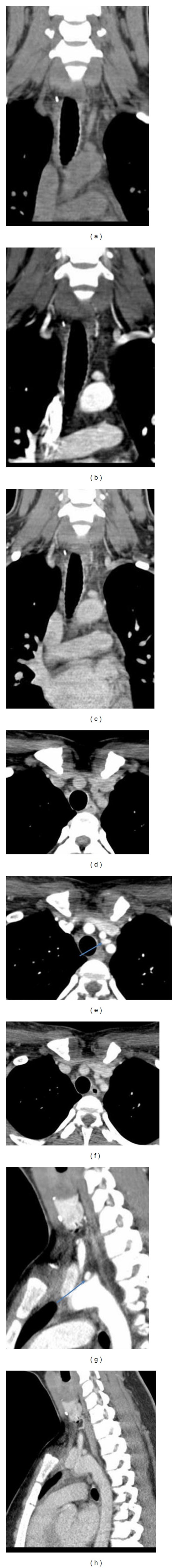
Coronal, axial, and sagittal views of 4DCT during 30, 60, and 90 seconds washout demonstrating hyperenhancement of ectopic parathyroid adenoma at 60 seconds.

On arterial phase images, a 4 × 11 × 6 mm hypervascular nodule was identified in the left side of the mediastinum, just above the aortic arch and between the left common carotid and left subclavian artery (Figure 1). On delayed images, there was rapid washout of contrast of the lesion.

On the basis of this 4DCT, the patient underwent a targeted exploration in the left mediastinum, and a 280 mg parathyroid mass was identified within the carotid sheath nestled between the take-off of the left subclavian artery and the carotid artery, posterior to the innominate vein. Postoperative calcium levels normalized and on followup iPTH dropped by half. Pathology showed that the 280 mg parathyroid mass was consistent with parathyroid adenoma. Interestingly, venous sampling from the second exploration before showed elevated intraoperative PTH levels within a similar location to where the occult adenoma was in the chest (Table 1).

## 3. Discussion

For patients with persistent primary hyperparathyroidism (PHPT), the identification of an occult lesion is key in decreasing morbidity associated with surgical exploration. Studies have shown increased rates of recurrent laryngeal nerve injury and permanent hypocalcemia in patients who underwent reoperation for persistent primary hyperparathyroidism [2, 3]. Ectopic locations for parathyroid adenomas include mediastinum, intrathyroid, intracarotid sheath, and intraneural sites [4]. For cases whereby sonography and sestamibi do not reproduce localizable lesions, evaluation with a cross-sectional imaging technique may be necessary. The decision to use 4DCT in the algorithm of persistent primary hyperparathyroidism is an important discussion as demonstrated by our case report.

Studies on 4DCT have shown higher sensitivity and specificity compared to those of sestamibi and ultrasound for identifying occult parathyroid adenomas. Chazen et al. identified 91% sensitivity and 93% specificity for lateralization of single gland disease using 4DCT, stratifying adenoma from four-gland hyperplasia [2]. Beland et al. identified 82% sensitivity and 92% specificity of 4DCT localization of adenomas in patients who had a history of failed surgery or unsuccessful localization on standard imaging [3]. The sensitivity specific range for 4DCT in other studies has been 70–88%, higher than that of sestamibi scanning (33–65%) and ultrasound (29–57%) [3]. The current consensus before proceeding with reoperation is that two concordant preoperative imaging studies should localize the hyperfunctional parathyroid tissue to the same anatomic region of the neck [3]. 4DCT is superior compared to sestamibi and ultrasound combined [3]. The advantage of 4DCT over MRI was illustrated in our case by the rapid washout of radiotracer from the left mediastinal adenoma, which was not picked up on MRI. The cost of performing 4DCT is higher than that of sestamibi and ultrasound for initial localization within the neck, and it is therefore not obtained unless non localization is proven on sestamibi and ultrasound [2].

MR imaging with contrast has been useful when sestamibi and US conflict; however, 4DCT may be more sensitive for detecting enhancing parathyroid lesions over MRI. Isointense images on T1 and T2 sequences on MRI may produce false-negatives as enhancement characteristics may resemble those similar to cervical lymph nodes. In comparison, 4DCT shows early enhancement pattern of parathyroid adenomas, different than lymph nodes, making it easy to differentiate from the two [3]. For pediatric patients with higher prevalence of nonspecific lymphadenopathy, the sensitivity of 4DCT in differentiating the two may be reason to consider 4DCT before MRI after ultrasound and sestamibi failed the first time.

Intraoperative venous sampling of PTH during four-gland exploration can be an adjunct to 4DCT in the lateralization of an adenoma. In our case, venous sampling taken at the time of the second surgical showed highest PTH in the areas of the left innominate vein and superior vena cava, coinciding with the left sided location of the occult parathyroid adenoma found on 4DCT in the mediastinum. We believe that the global elevation of intraoperative PTH from all sites is likely secondary to persistent PTH levels, as compared to the 100-fold gradient normally seen between venous samplings that confirms localization of the adenoma intraoperatively [5]. It may be favorable to utilize venous sampling during a neck exploration in order to confirm laterality of gland disease. Gawande et al. also concluded that when one of two preoperative imaging studies is unable to confirm lateralization of disease, intraoperative PTH measurement is essential in directing surgical excision of the adenoma [6].

Based upon our case report, we put forth an algorithm in minimally invasive direct parathyroidectomy for persistent primary hyperparathyroidism. For patients who have had nonlocalizing ultrasound and sestamibi, a four-gland exploration is warranted, whereby intraoperative PTH sampling should be used. If surgical exploration failed, then a diagnosis of ectopic location should be considered and proven with the use of 4DCT. The precision in which an imaging study can identify the lesion is paramount to minimally invasive parathyroid surgery. While the utility of 4DCT as a standard imaging technique has not outweighed the risks associated with increased radiation exposure, it is a highly sensitive and specific protocol for detection of ectopic disease. This will help to decrease morbidity and failure in the already operated neck and successfully treat PHPT in young patients through a minimally invasive approach.

## Figures and Tables

**Table 1:** Intraoperative PTH values via venous sampling during second surgical exploration.

Sample number	Location	PTH value
1	Left high internal jugular vein	200.8
2	Left mid internal jugular vein	207.0
3	Left low internal jugular vein	219.9
4	Left innominate vein	280.3
5	Right high internal jugular vein	241.1
7	Right low internal jugular vein	231.9
8	Right innominate vein	234.0
9	Superior vena cava	316.7
10	Right atrium	203.5
11	High IVC	140.8
12	Mid IVC	166.8
13	Low IVC	176.3
